# Decreased expression of the β_2_ integrin on tumor cells is associated with a reduction in liver metastasis of colorectal cancer in mice

**DOI:** 10.1186/s12885-017-3823-2

**Published:** 2017-12-06

**Authors:** Aitor Benedicto, Joana Marquez, Alba Herrero, Elvira Olaso, Elzbieta Kolaczkowska, Beatriz Arteta

**Affiliations:** 1 Department of Cellular Biology and Histology, University of the Basque Country, School of Medicine and Nursing, 48940 Leioa, Bizkaia Spain; 2 Department of Evolutionary Immunology, Institute of Zoology, Jagiellonian University, 30-387 Krakow, Poland

**Keywords:** Liver metastasis, Colorectal cancer, β_2_ integrin, LFA-1, ICAM-1, Immune response, Endothelial cells, Tumor microenvironment

## Abstract

**Background:**

Lymphocyte Function-Associated Antigen-1 (LFA-1; CD18/CD11a) is one of the main adhesion molecules used by immune cells to infiltrate the liver under inflammatory conditions. Recently, the expression of this integrin has also been reported on several solid tumors, including colorectal cancer. However, its functional role in the metastatic progression to the liver remains unknown. Using in vitro assays and an experimental orthotopic in vivo model of liver metastasis, we aimed to elucidate the role of tumor LFA-1 in the metastatic progression by means of the partial depletion of the β_2_ subunit of LFA-1, required for integrin activation, firm adhesion and signaling.

**Methods:**

To do so, we evaluated the effects of β_2_ reduction on the murine colon carcinoma C26 cell line on their pro-metastatic features in vitro and their metastatic potential in vivo in a mouse model of colon carcinoma metastasis to the liver.

**Results:**

The reduction in β_2_ integrin expression correlated with a slower proliferation, and a reduced adhesion and migration of C26 cells in an in vitro setting. Additionally, tumor cells with a reduced in β_2_ integrin expression were unable to activate the liver sinusoidal endothelial cells (LSECs). This resulted in a recovery of the cytotoxic potential of liver lymphocytes which is compromised by LSECs activated by C26 cells. This was related to the abrogation of RNA expression of inflammatory and angiogenic cytokines by C26 cells after their activation with sICAM-1, the main ligand of β_2_α_L_. Furthermore, in vivo tumor cell retention and metastasis were profoundly reduced, along with a decrease in the recruitment and infiltration of myeloid derived suppressor cells (MDSCs) and lymphocytes to the liver.

**Conclusion:**

Taken together, our findings uncovered the modulatory role for the tumor β_2_ subunit of the LFA-1 integrin in the metastatic progression of colorectal cancer to the liver by impairing activation of liver endothelium and thus, the local immune response in the liver. Besides, this integrin also showed to be critical in vivo for tumor cell retention, cytokine release, leukocyte recruitment and metastasis development. These data support a therapeutical potential of the integrin LFA-1 as a target for the treatment of colorectal liver metastasis.

**Electronic supplementary material:**

The online version of this article (10.1186/s12885-017-3823-2) contains supplementary material, which is available to authorized users.

## Background

Hepatic metastasis still remains as one of the most life-challenging aspects in the dissemination of cancer. The early retention of the tumor cells into a secondary organ, which leads to metastasis, might be due to the up-regulation in the expression of adhesion molecules and/or changes in their distribution [1, 2], enabling the adhesion and infiltration of metastasizing cancer cells in the target organ [1, 3]. In fact, the reciprocal interaction between liver sinusoidal endothelial cells (LSECs) and cancer cells through these adhesion molecules also triggers an acute inflammatory response [2, 4] which helps in the creation of a suitable microenvironment favoring the metastatic progression.

Lymphocyte Function Associated Antigen (LFA)-1 (CD11a/CD18 or α_L_β_2_) is a heterodimeric protein of the integrin family expressed on the surface of nearly all leukocytes [5] and recently described on a variety of tumor cells [1, 6, 7] including colorectal cancer cells [8, 9]. LFA-1 is the main ligand for intercellular adhesion molecule (ICAM)-1 [10, 11] to which the integrin binds with the highest affinity. Even though recent studies have shown a relationship between LFA-1 expression and the metastatic progression [12], up to date the functional role of this integrin in the development of liver metastasis is poorly characterized.

The liver is the main organ colonized during the progression of colorectal cancer patients, where LSECs constitute the first barrier cancer cells encounter and adhere to when invading the liver, function facilitated by the broad repertoire of adhesion molecules expressed on their surface [2]. Among others, the cell adhesion molecule ICAM-1 is constitutively expressed on LSECs and its expression is significantly increased during diverse inflammatory processes and at early stages of liver metastasis [13]. Additionally, the operating mechanisms used by immune cells to adhere to the liver endothelium and to infiltrate the organ afterwards are unique. This process in the liver involves different steps than those ones reported in the classical rolling-adhesion-extravasation paradigm. In some inflammatory scenarios, the direct adhesion based on LFA-1/ICAM-1 interaction was observed [14]. Interestingly, several solid cancers have shown a high expression of these two molecules including pancreatic cancer [2], and a decreased expression of LFA-1 on lymphoma cells has been correlated with a reduced invasion and metastases in vivo [15]*.* In line with these reports, we showed previously that LFA-1 expression correlates with the production of angiogenic factors by C26 cells, such as VEGF [12], as well as with an increase in the development of metastatic foci in the liver [12].

In addition, the local immune response developed in the liver during tumor infiltration determines the survival of cancer cells. In this organ, liver sinusoidal lymphocytes (LSLs) comprise the main population of immune cells, and develop an immune response during metastatic colonization. However, we have previously reported that tumor-activated LSECs decreased the cytotoxic potential of these lymphocytes towards C26 cells in vitro, mediated by the activity of mannose receptor (ManR) expressed on LSECs [4]. Furthermore, the previous stimulation of tumor cells with soluble ICAM-1 (sICAM-1) increased the activity of ManR on LSECs and further reduced the cytotoxic potential of LSLs once they have interacted with tumor activated LSECs [4]. Moreover, either the ManR blockage on tumor-stimulated LSECs or the neutralization of ManR stimulating factors derived from sICAM-1 activated tumor cells, such as Interleukin (IL)-1β inducing factors and Cyclooxygenase (COX)-2-dependent factors, restored the cytotoxicity of LSLs towards the cancer cells after their interaction with tumor-activated LSECs [4].

All these data led us to hypothesize that colon carcinoma cells could mimic the paradigm of leukocyte recruitment to the liver by means of the LFA-1/ICAM-1 pathway. Here, we assessed the effect of the reduced expression of the β_2_ subunit of the LFA-1 integrin during tumor progression of C26 colon cancer cells to the liver. Herein, we demonstrate that a decrease in LFA-1 β_2_ subunit expression limits the retention and the migratory potential of tumor cells in the liver and reduces the recruitment of immune cells into the organ leading to a diminution in the metastatic progression. This might be related to the activation of an inflammatory microenvironment triggered by tumor LFA-1 with endothelial ICAM-1. Thus, our results demonstrate that the full expression of LFA-1 integrin expressed on the surface of tumor cells facilitates the formation of liver metastasis during C26 colon carcinoma progression by initially driving the pro-tumoral activation of LSECs, and inducing the infiltration of the liver by immune cells with regulatory functions. These results point out LFA-1 as a potential therapeutic target in the treatment of hepatic metastatic disease.

## Methods

### Animals

Eight weeks old male Balb/c mice were obtained from Charles River (Barcelona, Spain). Housing, care, and experimental conditions were carried out in conformity with institutional guidelines and national and international laws for experimental animal care. The animals were fed a standard chow and had access to water ad libitum. All the proceedings were approved by the Basque Country University Ethical Committee (CEID) in accordance with institutional, national and international guidelines regarding the protection and care of animals use for scientific purposes.

### Cancer cell lines

All in vitro and in vivo experiments were conducted using the murine C26 colon adenocarcinoma (C26) cell line (also known as MCA-26, CT-26) syngenic with Balb/c mice and purchased from ATCC (LGC Standards S.L.U. Barcelona, Spain). The C26 cell line was genetically modified to partially deplete the expression of the β_2_ subunit of the LFA-1 receptor (named β_2_-C26) by Innoprot S.L. (Zamudio, Spain). The cDNA sequence corresponding to Itgb2, with accession number NM:008404, was introduced in the siDESIGNER CENT from Dharmacon (Lafayette, CO), and the sequences siItgb2–1: tcggaaggtgttggataa, siItgb2–2: ggtgaaaacgtatgagaaa, and si Itgb2–3: ctgcatgtccggaggaaat were selected. The three sequences were cloned in the vector containing pSUper-Purofor, a vector system for expresson of shRNA induction of Puromycin resistance (Oligoengine; WA, USA) to produce the corresponding shRNA. The pSuper-RNAi system provides a mammalian vector that directs intracellular synthesis of siRNA-like transcription. The resulting transcript of the recombinant vector is a predicted shRNA. The transcript is quickly cleaved to produce a functional siRNA. After 48 h transfection with 1 μg of each plasmid containing the sequence to obtain either of each shRNA, the tumor cells were cultured in the presence of puromycin (10 μg/ml) to obtain isolated clones. In some experiments a pool of the transfected cells were used. After 2–3 weeks of culture in the presence of puromycin 24 clones/shRNA tested were selected. Then, those clones were amplified and the six with lower expression were selected for stable lines generation. When the amount of 1,2 × 10^6^ cells was obtained, a RNA amplification for β_2_ integrin allowed the selection of the clone with the lower β_2_ expression for experimentation. The primers used for amplification were Itgb2 F: ATGTGGGCCCACACTCACTGC and Itgb2 R: TTAACAAAAGGCAGCACCGT. The clone was cultured under standard conditions in RPM-1640 supplemented with 10% heat-inactivated fetal bovine serum (FBS), penicillin (10,000 U/ml), streptomycin (10.000 μg/ml) and amphotericin B (25 μg/ml) supplemented with 10 μg/ml Puromycin.

### Culture of primary LSEC

The isolation and culture of mouse LSECs have been described elsewhere [4, 16]. Purified LSECs were cultured on 1 mg/ml collagen type I from rat tail – 0′25 ml/cm^2−^ (Sigma-Aldrich, St. Louis, MO, USA) coated tissue culture plates at a concentration of 3′5 × 10^5^ cell/cm^2^ in RPMI-1640 supplemented with 5% FBS, antibiotics and antimycotics. LSECs were incubated at 37 °C, 5% CO_2_ for at least 2 h before experimental procedures. Cultures of LSEC were pre-activated for 16 h with β_2_-C26 cells or C26 cells prior to different analyses.

### Cancer cell adhesion assay

Either C26 or β_2_-C26 cells previously labeled with 25 μM CFSE (Life Technologies Inc.; MD, USA) at 37 °C were added to LSECs cultures or collagen type I coated wells at a concentration of 2 × 10^5^ cells/ml. In additional experiments β_2_-C26 pool, or β_2_-neutralizing antibody (1 μg/10^6^ cells; BD Pharmingen, Madrid, Spain) pretreated C26 were also added to collagen type I coated wells. After an incubation of 30 min, total fluorescence was measured by using Ascent Fluoroskan (Labsystems S.A.C., MA, USA). Then, fluorescence emitted by adhered cells was measured after an extensive washing with culture medium to remove non adherent cells. The percentage of tumor cell adhesion was calculated after background subtraction as follows:

% adhesion = (fluorescence emitted by adhered cells × 100)/ total fluorescence.

In some experiments, tumor cells were treated with blocking antibodies against CD11a (Clone M17/4) after tumor cell activation with sICAM-1 before their addition to LSECs culture. In others, tumor cells were pre-treated with antibodies against CD11b (Clone EPR1344) and polyclonal CD11b/c, and LSECs were pre-treated with blocking antibodies against CD106 (VCAM-1) Clone 429, at a concentration of 1 μg/ml for 45 min, prior to the addition of the tumor cells.

### Migration and transendothelial migration assay

The migration assay was carried out on a modified Boyden chambers. Briefly, either C26, β_2_-C26 cells, β_2_-C26 pool, or β_2_-neutralizing antibody (1 μg/10^6^ cells; BD Pharmingen, Madrid, Spain) pretreated cells were seeded onto type I collagen (1 mg/ml) -coated 8 μm-diameter pore Transwell inserts (Greiner Bio-one, Germany) and a total of 2 × 10^4^ tumor cells in 100 μl of cell culture medium supplemented with 1% FBS and antibiotics were added to the upper chamber. In some experiments, tumor cells were allowed to adhere and expand before addition of sICAM-1 (200 ng/ml) (Life Technologies Inc). Then, they were allowed to migrate for 18 h before processing and quantification. For transendothelial migration, 2 × 10^5^ LSECs were seeded and allowed to adhere and spread for 2 h before tumor cell addition. Then, C26, β_2_-C26 cells were allowed to migrate and transmigrate for 42 h, respectively, and quantified after 4% formalin fixation and crystal violet staining (Sigma-Aldrich). Data are expressed relative to the number of parental C26 cells migrated under basal conditions, in both migration and transmigration studies.

### Viability assay

PrestoBlue Cell Viability Reagent® (Life Technologies Inc.) was used for quantification of viable tumor cells following manufacturer instructions. After 3 h (time 0) and 48 h of culture, C26 cells and β_2_-C26 cells viability was measured by adding Presto blue reagent for 90 min. Absorbance was measure with the Ascent Multiskan (Labsystems). Increase in cell viability after 48 h were compared with the cell viability at time 0.

### Analysis of cell cycle and number of cell divisions

A quantity of 5 × 10^5^ C26 or β_2_-C26 cells were collected and washed with PBS. After fixation in 2% formaldehyde containing PBS, the cells were resuspended in 500 μl of FxCycle™ PI/RNAse Solution (Life Technologies Inc.) and incubated for 30 min. Then, differences in PI labeling were assessed by FACS (EPICS S Elite, Beckman Coulter, Brea, CA, USA) and analyzed using the Weasel free software (WEHi, Parkville, VIC, Australia) with a specific cell cycle protocol. For quantification of n° of cell divisions, tumor cells were labeled with CFSE. Non divided cells were represented by those fixed before culturing. The remaining cells were re-suspended to 5 × 10^5^ cells, and cultured for 48 h. Cells were then collected and fixed and total emitted florescence was measured by FACS and analyzed by Weasel free with a specific CFSE protocol. The fluorescence emitted by these cells, fixed at time 0, is considered to be the maximal amount of fluorescence measured in the assay and represents those cells which have not suffered any division. Thus, the number of cell cycles can be quantified by a decrease in the fluorescence emitted by a group of cells at a specific time point.

### Endocytosis and antigen processing assay

ManR activity was measured by LSEC incubation with FITC-labeled mannan (10 μg/ml) (Sigma-Aldrich, IL, USA) for 2 h. The mannan uptake was quantified by Ascent Fluoroskan (Labsystems) and expressed as the percentage of internalized mannan respective to total added amount. Next, processing of DQ-ovalbumin (Life Technologies Inc.) was measured by 30 min incubation of LSECs with the ligand. After excess of DQ-ovalbumin was removed, its processing was quantified as the increase of fluorescence respective to the Initial one.

### Real time-PCR analysis

Cell lysates of C26 and β_2_-C26 cells were obtained after their previous stimulation with sICAM-1. Total RNA was extracted using PureLink® RNA Mini kit (Life Technologies Inc.), according to the manufacturer’s instructions. RNAse-free DNase I was used to prevent DNA contamination. RNA concentration was assessed by absorbance at 260 nm using a NANO DROP spectrophotometer (ND-1000, Thermo Scientific, Rockford, IL), and the purity of the samples was estimated by the OD ratios (A260/A280, ranging within 1.8ROP2). Reverse transcription (RT) was performed in a 20 ml reaction volume with 2 lg of total RNA treated with 25 mM MgCl2, PCR buffer 103, 100 mM dithiothreitol (DTT), 0.5 ll of Oligo(dt16), 50 U multiscribe reverse transcriptase, 40 U RNase inhibitor and 40 mM dNTP to synthesize first-strand cDNA. Reaction system was incubated at 25 °C for 10 min (primer annealing), 42 °C for 15 min (synthesis) and final temperature of 4 °C, and resulting cDNA was stored at 220 °C. The resulting cDNA was subjected to RT-qPCR for the evaluation of the relative expression levels of b-actin (as an internal control). Gene-specific amplification was performed using ABI 7900HT, a RT-qPCR machine (Life technologies, Grand Island, NY) that measures binding of SYBR Green I to double- stranded DNA. Each sample was tested with a no template control for each pair of oligonucleotide primers to control contamination or primer dimer. Each experiment was repeated at least three times using cDNA samples from separate RT reactions. The reactions were performed in a total volume of 10 ll that contained the following: 35 ng cDNA that was synthesized as described above, 5 ll of SYBR Green master mix (Life technologies) and 200 nM of each pair of oligonucleotide primers. The amplification was performed as follows: an initial step at 95 °C for 10 min, followed by 45 cycles of 95 °C for 30 s and 60 °C for 60 s. Regression curves were calculated for each sample, and the amplified sample were calculated for each from the threshold cycles using the instrument. The following primers for RT-PCR analyses were used:

Itgb2: forward 5′-ATGTGGGCCCACACTCACTGC-3′ and reverse 5′-TTAACAAAAGGCAGCACCGT3′;

VEGF: forward 5′-TGTACCTCCACCATGCCAAG-3′, reverse 5′-ACTTGATCACTTCATGGGACTTCT-′3′;

COX-2: forward 5′-TGCACTATGGTTACAAAAGCTGG-3′; reverse 5′-TCAGGAAGCTCCTTATTTCCCTT-3′.

### LSLs isolation and tumor cytotoxicity assay

LSLs were obtained by means of liver perfusion with PBS-0.1 mM EDTA and Lympholite M (Cederlane, Canada) gradient centrifugation as previously described [4]. For assessment of cytotoxic activity of LSLs towards C26 cells, lymphocytes were allowed to interact with activated and not activated LSECs for 24 h. The activation of LSECs was induced by incubation with either C26 cells or β_2_-C26 cells. Then, LSLs were collected and added to target tumor cells at a ratio of 5:1 effector/target cells. After 24 h of co-incubation, tumor cell viability was estimated by the Presto Blue assay (Life Technologies Inc.). Data were expressed as 100- % C26 viability respective to untreated cells.

### Early retention of cancer cells in the liver and experimental development of hepatic metastasis

For tumor cell retention and hepatic metastasis, 2 × 10^5^ of either C26 cells or β_2_-C26 cells were intrasplenically (i.s.) injected into anesthetized mice as previously described [4]. For retention studies, tumor cells were firstly labeled with CFSE as described above, and mice euthanized 24 h later. Livers were removed and embedded in OCT (Tissue-Tek®, The Netherlands) and frozen in dry-ice. For hepatic metastasis, mice were inoculated with C26 cells, β_2_-C26 cells, β_2_-C26 pool cells or neutralizing β_2_-antibody pre-treated C26 cells injected i.s. into anesthetized mice. Then, mice were sacrificed 14 days after tumor cell inoculation, livers were collected, fixed in zinc-fixative solution (Sigma-Aldrich, MO, USA) and paraffin embedded for histological analyses after H&E staining or embedded in OCT and frozen in dry-ice for fluorescence immunohistochemical studies. Tumor occupied area was quantified in three10 μm thick sections per liver, separated by 500 μm from each other. The total tumor burden was calculated as the sum of the area of each of the metastatic foci in 100 mm^2^ of liver section. Additionally, the number of foci within a specific size range was also calculated. At least 5 mice per group were used per each experiment and each one was performed 3 times for those experiments using the partially silenced clone and 4 mice for those experiments using the pool of partially depleted cells and cells pre-treated with anti β_2_ integrin antibody.

### Immunohistochemical analysis

Frozen liver sections were analyzed for the quantification of different immune cell populations, 24 h and 14 days after the tumor cell injection. The quantification of CD4^+^, CD8^**+**^, CD11b^+^ and Ly6G^+^ (Gr1^+^) cell numbers was carried out in 3 different sections per mice, and At least 5 mice per group were used per each experiment and each one was performed 3 times. Anti-CD4 monoclonal antibody (Life Technologies, Inc.), anti-CD8 monoclonal antibody and anti-CD11b (both from Abcam; Cambridge, UK), and anti-Ly6G antibody (Novus Biologicals; CO, USA) were used. After blocking and incubation with specific primary antibodies, surface molecules were detected by the use of secondary antibodies conjugated either with Alexa-488 or Alexa-594.

### Statistical analyses

Data are expressed as mean ± standard deviation (SD) of three independent experiments. Statistical analysis was performed using SPSS version 13.0 (Professional statistic, IL, USA). Individual comparisons were performed using two-tailed, unpaired Student t test. Differences were considered to be significant for *p < 0.05 and **p < 0.01.

## Results

### Reduced expression of β_2_ integrin in transfected C26 cells

**Fig. 1:**
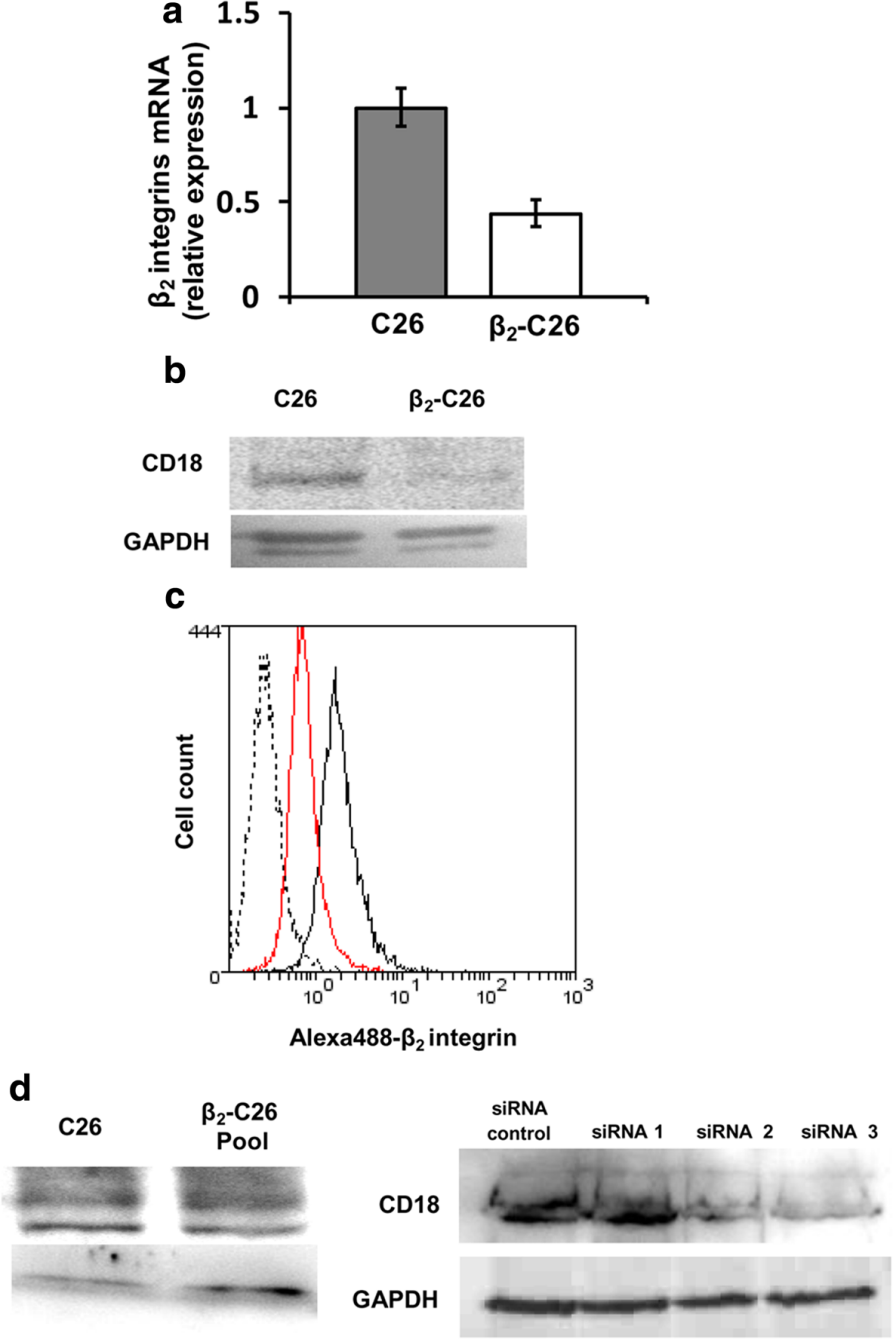
Analysis of the expression of β_2_-integrin on C26 and β_2_-C26 cells. Lysates of unmodified (wild type) and β_2_-depleted C26 cells were collected for RNA (**a**) and protein (**b-c**) expression levels. **a** For β_2_ integrin RNA analysis by RT-PCR the following primers were used: forward ATCCTGACCCCCAATGATGG, reverse 5’CGGATGGGTAGTCGAACTCA. GAPDH was used as an internal control (house keeping gene) (**b**) Protein lysates were obtained from 1 clone cell line clones as described in Material and methods. At the protein level, the β_2_ integrin was detected by Western Blotting applying specific antibodies recognizing the β_2_ subunit of the integrin. **c** Tumor cells were incubated in the presence of specific antibodies for the integrin β_2._ Then, cells were subjected to FACS analysis after incubation of Alexa-488 antibody. The black line represents C26 cells, the red line represents β_2_-C26 cells and dash line represents the negative control. **d** Protein lysates were obtained from a pool of stably transfected C26 (*left*). Additionally, protein lysates were obtained from C26 tumor cells transiently transfected either with a control siRNA or three siRNAs specific for β_2_ integrin (*right*). At the protein level, the β_2_ integrin was detected by Western Blotting applying specific antibodies recognizing the β_2_ subunit of the integrin

A stable cell line with a reduced expression of β_2_ integrin was established and routinely tested for the level of β_2_ integrin expression at RNA level (Fig. 1a) and at protein level (Fig. 1b) by real time-PCR and western blot analyses, respectively. Additionally, the levels of β_2_ integrin were assessed by FACS analysis (Fig. 1c) and found to be expression decreased in the stable C26 cell line, as observed by FACS, RT-PCR and Western blot. Additionally, the levels of β_2_ expression in a β2-depleted C26 pool and in cells transiently transfected with the siRNAs were also quantified (Fig. 1d).

### Reduced activity of β_2_ integrin on tumor cells decreases the metastatic development in the liver

**Fig. 2:**
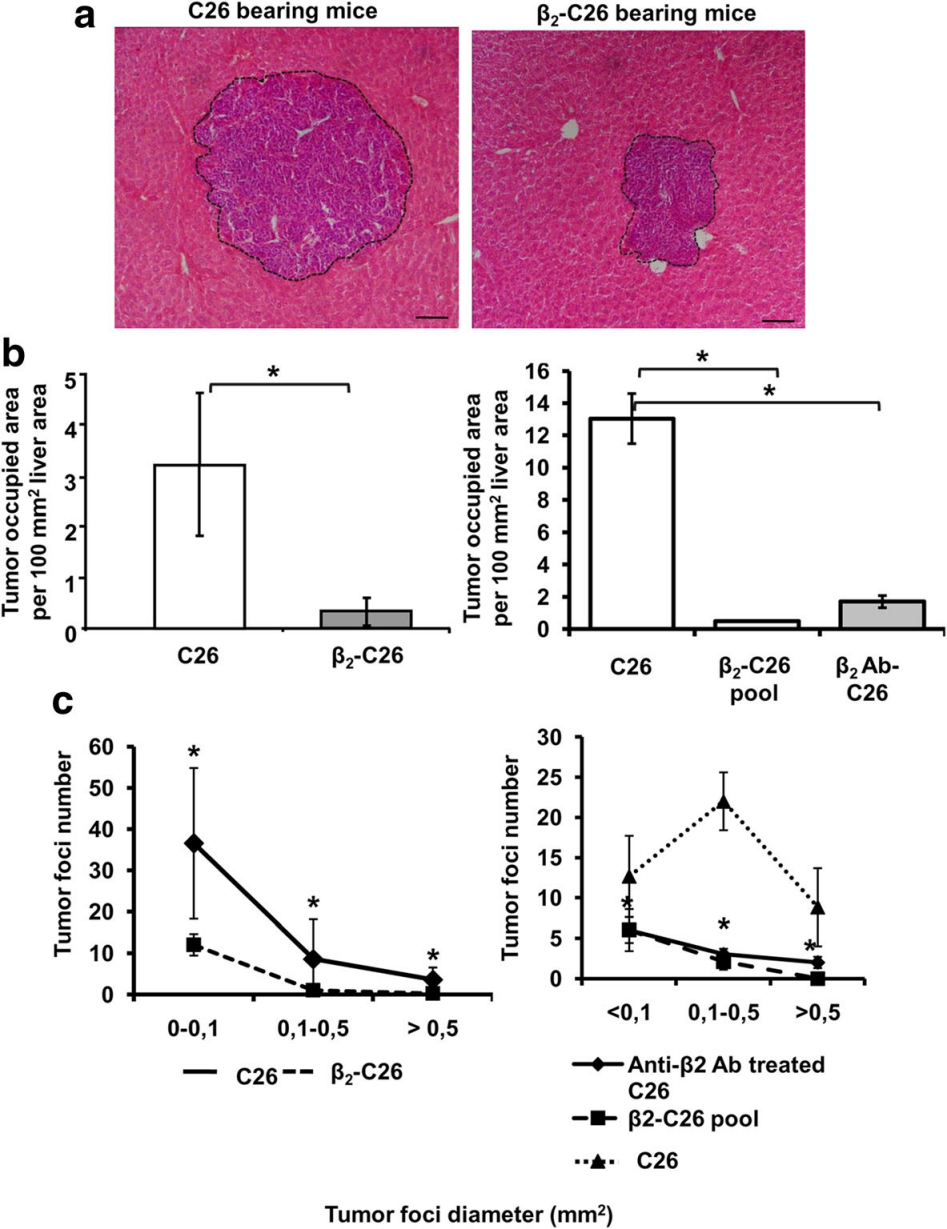
Effect of the partial deficiency of the integrin subunit β_2_ on C26 colon carcinoma cells on tumor metastasis to the liver. Mice were sacrificed 14 days after either C26, β_2_-C26 cells i.s. inoculation, and metastatic development was quantified in paraffin embedded liver sections. In some experiments the mice were inoculated with a pool of β_2_-C26 cells or with C26 pre-treated with a neutralizing antibody for β_2_ integrin. **a** H&E staining showing a reduced size of metastatic foci in animals injected with β_2_-C26 cells versus C26 cells. **b** The area of liver occupied by the C26 cells or β_2_-C26 cells (*left*) and by C26, a pool of β_2_-C26 cells or by C26 pre-treated with a neutralizing antibody for β_2_ integrin (*right*). Total tumor burden was quantified as the area occupied by the tumor per 100 mm^2^ of liver area. **c** Tumor foci were classified by their size and their number quantified per liver tissue section. Data obtained from mice inoculated with C26 cells or β_2_-C26 cells are shown in the left and by C26, a pool of β_2_-C26 cells or by C26 pre-treated with a neutralizing antibody for β_2_ integrin in the right. Data are mean values ± SD from three different experiments (*n* = 15). Changes were considered statistically significant at * *p* < 0.05 and **p < 0.01. Scale bar 100 μm

To establish the metastatic potential of β_2_-C26 cell, the cells were i.s. Inoculated into Balb/c mice. A second group of animals were inoculated with wild type C26 as control. Either cell type induced metastasis in the liver 14 days after inoculation (Fig. 2a). However, the colonization of the liver by β_2_-C26 cells was significantly reduced comparing to that of parental C26 cells. The area occupied by tumor in the liver of mice bearing C26 cells (Fig. 2b left) was significantly reduced. These results show the decrease in the metastatic potential of tumor cells after the partial depletion of β_2_ integrin subunit. To further confirm that the observed reduction was not due to the absence of clonal variability but to the depletion of the β_2_ integrin subunit, additional experiments were carried out by inoculating a pool of stably transfected cells or C26 cells pre-treated with β_2_ integrin neutralizing antibody. Under this conditions, liver colonization was also reduced as a result of the absence of integrin β_2_ (Fig. 2b right). As shown in Fig. 2c, the various experimental conditions where the β_2_ integrin subunit was depleted caused a decrease in the tumor burden as a result of a reduction in the number of foci of each size.

### Reduced expression of tumor β_2_ integrin decreases tumor cell adhesion in vitro

**Fig. 3:**
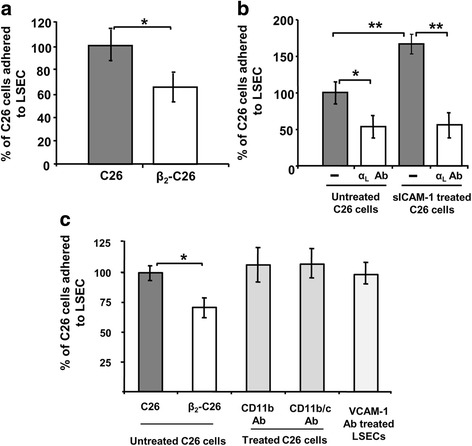
Modulation of the adhesive potential of C26 cells by reduced expression of β_2_ integrin. The ability to adhere to liver endothelial cells (LSECs) of C26 and β_2_- C26 cells was tested with adhesion assays. **a** C26 and β_2_-C26 cells were added to cultures of primary isolated LSECs and allow to adhere for 30 min before quantification of cell adhesion. **b** In subsequent experiments, C26 cells were pre-activated with sICAM-1 (200 ng/ml). Before sICAM-1 activation some tumor cells were pre-treated with neutralizing antibodies for the subunit α_L_ of the integrin. **c** In order to rule out other adhesion molecules present on the surface of tumor cells or LSECs, C26 cells were pre-treated either with CD11b or with CD11b/c, and LSECs were pre-treated with VCAM-1 antibodies before the addition of tumor cells to LSEC cultures. Data are mean values ± SD from three different experiments. Differences were considered statistically significant at *p < 0.05 and **p < 0.01

LSECs are the first cell type C26 cells encounter when entering the liver. Thus, the adhesion of tumor cells to the endothelium is believed to constitute a limiting step during early stages of metastatic progression. In order to analyze the effect of a reduced expression of the β_2_ integrin on tumor cells in the adhesion to LSECs, we quantified the adhesion of murine colon carcinoma cells C26 (grey bars) and β_2_-C26 (white bars) cells to LSECs (Fig. 3a). To do so, CFSE-labeled tumor cells were added to monolayers of primary LSECs. As shown in Fig. 3a, the reduced expression of β_2_ integrin diminished by 35% the amount of tumor cells adhered to endothelial cell monolayers. Additionally, to confirm the involvement of LFA-1, C26 cells were treated with a neutralizing antibody recognizing CD11a, the α_L_ subunit of the integrin, prior to their addition to LSEC cultures. Cell adhesion of C26 (grey bars) was affected by either the partial depletion of β_2_ (Fig. 3a) or the neutralization of α_L_ subunit (Fig. 3b). Interestingly, these antibodies also abolished the increase observed in the adhesion of C26 tumor cells after activation by sICAM-1 (Fig. 3b). On the contrary, no significant effect was noted when other ICAM-1 ligands (CD11b and CD11c) were blocked on tumor cells by neutralizing antibodies or when the ligand for the β1 integrin VLA-4, VCAM-1, was neutralized on LSECs (light grey, Fig. 3c). Moreover, the adhesion to immobilized sICAM-1 was decreased for β_2_-C26 cells (Additional file 1A).

### Partial deficiency in β_2_ integrin reduces cancer cell migration through LSEC monolayers and collagen

**Fig. 4:**
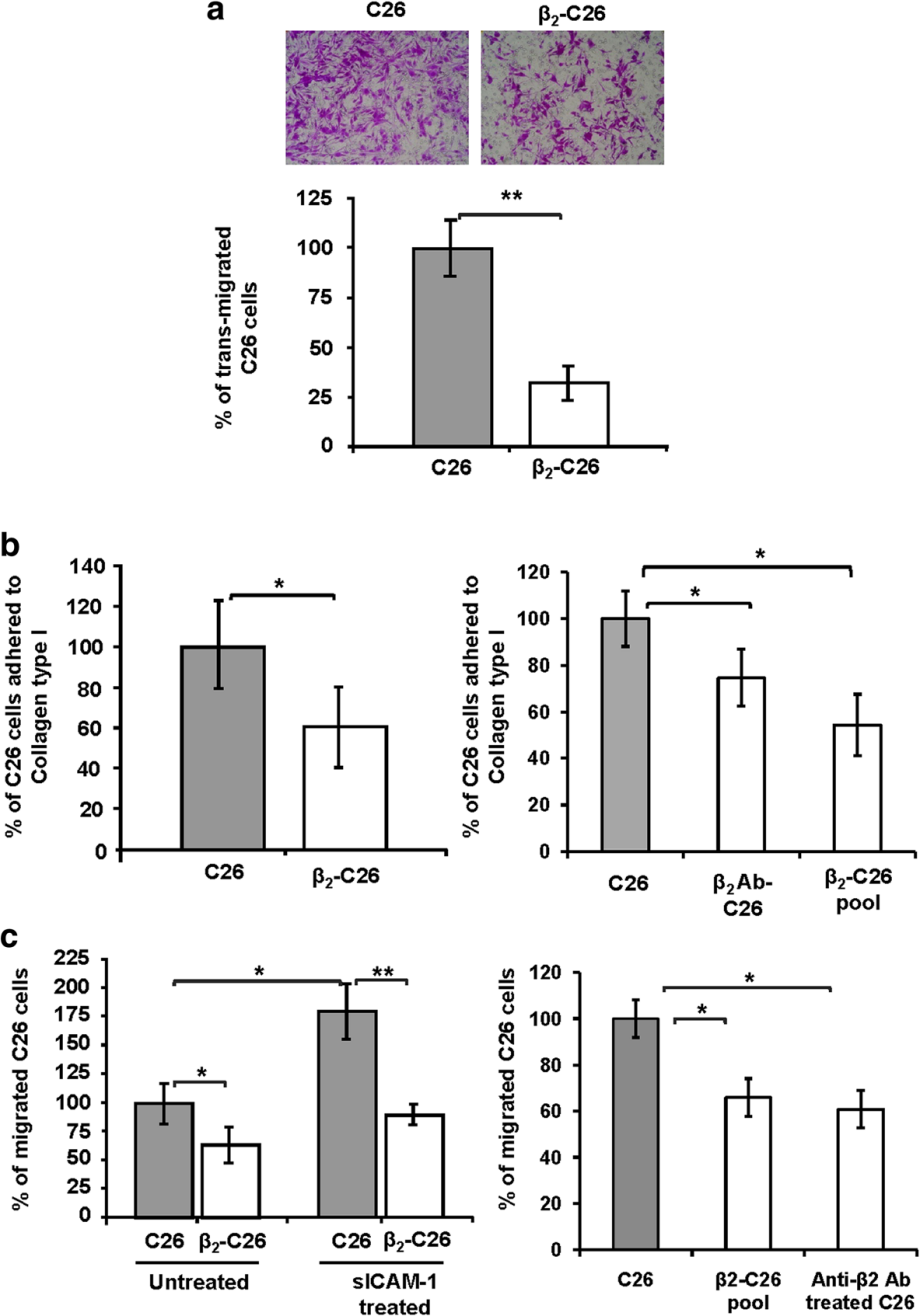
Reduced β_2_ integrin expression reduces migratory potential of C26 cells through LSEC, and adhesion to and migration through collagen type I. The ability of tumor C26 and β_2_-C26 cells to migrate across LSEC monolayers (**a**), and the ability of C26 cells or β_2_-C26 cells (*left*) and that of C26, a pool of β_2_-C26 cells or C26 pre-treated with a neutralizing antibody for β_2_ integrin (*right*) to adhere to a layer of collagen type I (**b**), and to migrate through collagen type I (**c**) was quantified. Transmigration and migration studies were carried out in modified Boyden chambers. Representative pictures of C26 cells transmigrated through the LSEC are shown in **a**. Migrated cells are expressed as the average number of cells that migrated per 20× field. Data are mean values ± SD from three different experiments. Differences were considered statistically significant at *p < 0.05 and *p < 0.01

Following adhesion to the endothelial cells in the sinusoids, tumor cells must migrate across a layer of LSECs, and then through the extracellular matrix (ECM) in the space of Disse, which is composed mainly by collagen type I. To assess the role of this integrin in these processes, we first analyzed the migratory potential of C26 cells through a monolayer of LSECs. After 42 h, a 3-fold decrease in trans-migrated tumor cells was observed for those with a reduced expression of β_2_ integrin compared to unmodified C26 cells (Fig. 4a). Secondly, we quantified the adherence of C26, β_2_-C26 cells, β_2_-C26 pool cells and anti-β_2_ pre-treated C26 cells to collagen type I by adding the tumor cells to a layer of collagen type I. Figure 4b left reveals a 40% reduction in the adhesive potential of β_2_-C26 compared to unmodified C26 cells, and of 30 and 45% for β_2_-C26 pool cells and anti-β_2_ pre-treated C26 cells respectively (Fig. 4c right). In addition, the adhesion of tumor cells, transiently transfected with a control siRNA and three different siRNAs, to collagen type I confirmed the siRNA 3 as the one with the highest silencing efficiency (Additional file 1B). Furthermore, the pre-treatment of either C26 or β_2_-C26 cells with a neutralizing antibody for β_1_ integrin showed no implication of this integrin in the reduction in adhesion observed after depletion of β_2_ integrin (Additional file 1C). Finally, the potential of C26 and β_2_-C26 cells to migrate through collagen I layer was tested (Fig. 4c left). The migration of β_2_ deficient cells was reduced by 25% (Fig. 4c left). Furthermore, the addition of sICAM-1 to the cultures increased 75% the number of migrated C26, while a lack of such response was observed when β_2_ was partially inactivated in the tumor cells activated by sICAM-1 (Fig. 4c left). As shown for the adhesion to collagen type I of β_2_-C26 cells, the pool of transfected C26 cells with a decreased expression of β_2_ integrin and those C26 cells pre-treated with neutralizing antibodies for the for β_2_ subunit also showed a reduced adhesive potential to the extracellular matrix protein (Fig. 4 right). Additionally, neutralization of β2 integrin subunit in MC38 colon carcinoma showed similar effects in metastatic, adhesive and migratory potential of the tumor cells (Additional file 2A-C).

### Partial deficiency on β_2_ integrin reduces proliferation in C26 cells

**Fig. 5:**
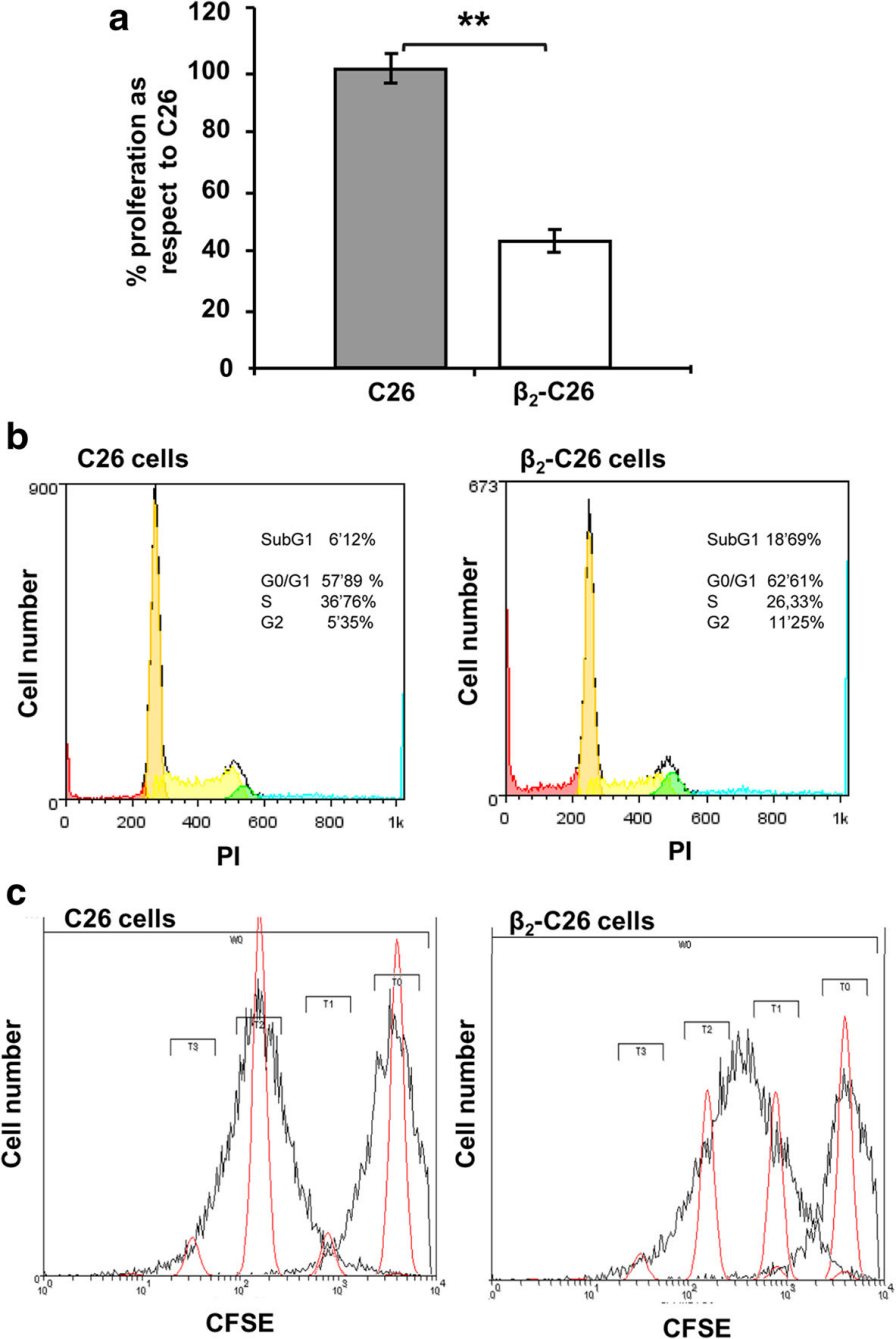
Decreased β_2_ expression on C26 cells modulates their proliferative activity*.* **a** Cell viability was assessed by the Presto Blue assay after 48 h of cell culture. **b** Cell cycle analyses were carried out by measuring the DNA content by flow cytometry of tumor cells stained with PI. **c** The number of divisions undetaken by tumor cells was quantified by CFSE assay. Control cells (C26) or β_2_-C26) were fixed immediately after CFSE- labeling to show fluorescence emitted by non-divided cells (T0), that is, at time 0 before cells were allowed to divide. The remaining tumor cells were further incubated for 48 h under normal culture conditions. Cells that underwent one cell division are gated in T1 and further constitutive numbers reflect the number of cell divisions (T2-T3). Cells included in each gate are represented by red peaks. Fluorescence data are mean values ± SD from three different experiments. Changes were considered statistically significant at * *p* < 0,05

In order to assess the role of β_2_ in the proliferation of colon cancer cells, firstly we evaluated in vitro viability of β_2_-C26 vs. C26 cells after 48-h incubation. A 2-fold reduction was observed in the number of β_2_-C26 tumor cells compared to that of unmodified C26 cells (Fig. 5a). To verify if this resulted from an interference with the cell cycle, we assessed the amount of cells in each phase of the cell cycle reporting a slight, but not significant, increase in the number of cells in G0/G1 and G2/M and also, a slight decrease in the number of cells in S (Fig. 5b). It is noteworthy, that cells in subG1 were increased by 3-fold. Then, we quantified the number of cycles undergone by the tumor cells within 48 h of their culture in comparison to cells at time 0 (T0 - first red peak) (Fig. 5c). As shown, the pattern of cell divisions was slightly, although not significantly, altered in β_2_-C26 tumor cells, since the number of cells in T3, representing those which have undergone 3 divisions, were lower. Besides, the number of β_2_-C26 tumor cells that divided only once (T1) or twice (T2) was higher than for C26 tumor cells (Fig. 5c).

### Tumor LFA-1 mediates ManR activation on LSEC

**Fig. 6:**
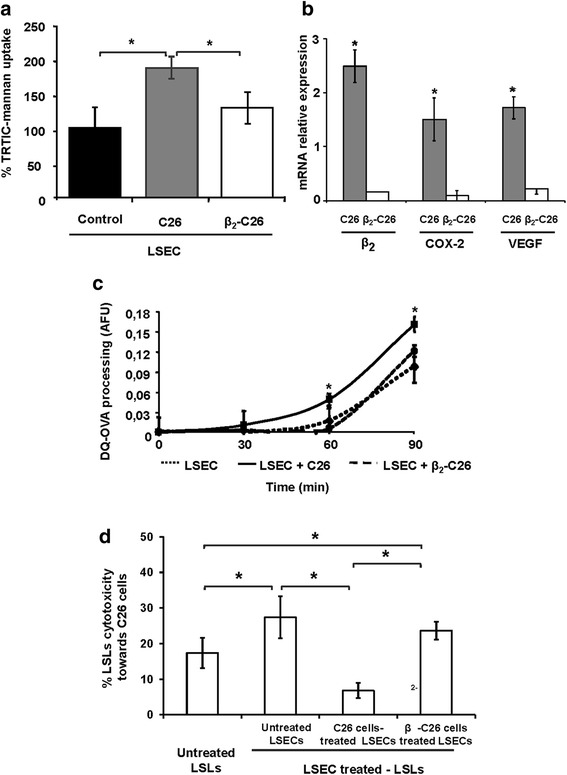
The potential of C26 cells to activate LSECs is mediated by tumor LFA-1 expression*.* Uptake of TRITC-labeled mannan (**a**) after stimulation with either C26 cells (*grey bar*) or β_2_-C26 cells (*white bar*) was quantified. Data are presented as % of internalized uptake by LSECs activated by either C26 or β_2_-C26 cells compared to that of LSECs cultured alone. **b** The expression of genes involved in the ability of C26 to altered Mannose receptor upregulation after ligation of LFA-1 with endothelial ICAM-1 was analyzed by quantitative PCR in tumor cell lysates after sICAM-1 (200 ng/m) stimulation. Data of RNA expression are presented as C26 cells gene expression relative to sICAM-1 activated C26 cells gene expression (*dark bars*) and β_2_-C26 cells gene expression relative to sICAM-1 activated β_2−_C26 cells gene expression (*white bars*). **c** DQ-ovalbumin processing by LSECs (*short dash line*) was quantified after stimulation with either C26 cells (*continuous line*) or β_2_-C26 cells (*long dash line*). Data are presented as AFU -arbitrary fluorescence units. Data are mean values ± SD from three different experiments. Changes were considered statistically significant at *p < 0.05. **d** Activation of LSECs by C26 cells with partial expression of β_2_integrin does not affect lymphocyte cytotoxic activity towards C26*.* Liver sinusoidal lymphocytes (LSLs) were incubated for 24 h with either untreated LSECs, or LSECs activated with C26 or β_2_-C26 cells. Then, LSLs were transferred to C26 cultures and co-incubated for another 24 h. Subsequently, their cytotoxic activity towards C26 cells was measured by the Presto Blue assay. Basal untreated LSLs (left bar) were used to quantify basal cytotoxic activity of LSLs towards C26 cells. Data are mean values ± SD from three different experiments. Changes were considered statistically significant at *p < 0.05

We tested ManR activation status on LSEC by means of FITC-labeled mannan uptake, a well-known ligand of ManR. This process is known to be altered on LSECs after direct endothelial ICAM-1/tumor LFA-1 interaction and abolished after the binding of those two adhesion molecules is blocked [4]. As shown in Fig. 6a, while the uptake of mannan by LSECs was increased after being co-cultured with C26 cells, no significant upregulation was observed when β_2_-C26 cells were added. Then, the ability to process an antigen was measured by quantifying the fluorescence emitted by degraded DQ-ovalbumin, after its uptake by ManR expressed on LSEC (Fig. 6b). The intracellular processing of DQ-ovalbumin by LSECs was increased after their co-culture with wild-type C26 but not with β_2_-C26 cells (Fig. 6b). Moreover, the increase in ManR-mediated expression of Vascular Endothelial Growth factor (VEGF) and COX-2 genes by the tumor cells in response to sICAM-1 stimulation was abrogated in β_2_-C26 cells (Fig. 6c).

### LSLs cytotoxic activity towards C26 is down-modulated by LSECs stimulated with by β2-C26

To evaluate the effects of tumor activated LSECs on the cytotoxic activity of LSLs towards C26 cells, LSLs were co-cultured with LSECs previously activated by cocultivation with either C26 or β_2_-C26 tumor cells. Then, LSLs were collected and transferred to C26 cultures and their cytotoxicity towards tumor cells was assessed. As shown in Fig. 6d, the contact of LSLs with resting LSECs increased their cytotoxic activity towards C26 cells, however, this cytotoxicity was reduced when LSLs were in contact with LSECs previously activated by C26 cells. On the contrary, the cytotoxic potential of LSLs towards tumor cells was recovered when LSECs were activated with β_2_-C26 cells (Fig. 6d).

### Decreased expression of tumor LFA-1 impairs early retention of cancer cells in the liver

**Fig. 7:**
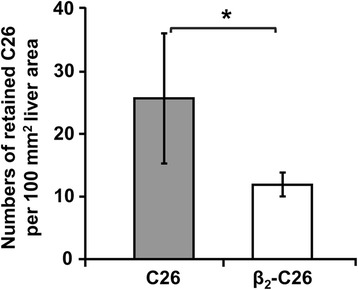
β_2−_integrin reduced expression prevents C26 cell retention in the liver. The retention of fluorescently labeled-C26 (*grey bar*) or β_2_-C26 cells (*white bar*) in the liver was quantified 24 h after i.s. inoculation of the tumor cells

The retention of cancer cells in the target organ is a key step for the metastatic development. To quantify retention of tumor cells in vivo, mice were i.s. injected with tumor cells and sacrificed 24 h later. A 2-fold reduction in tumor cell numbers retained in the liver was observed in the livers of mice injected with β_2_-C26 cells (Fig. 7), in line with the previous in vitro results.

### Decreased expression of tumor β_2_ integrin correlates with a reduced recruitment of immune cells at early stages of metastasis

**Fig. 8:**
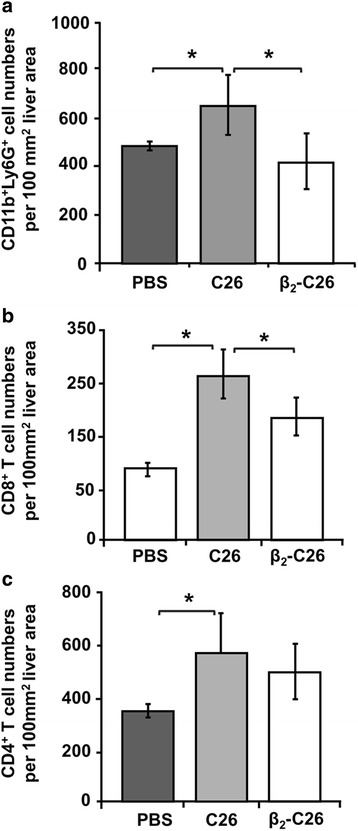
The reduced expression of tumor β_2_ integrin limits the infiltration of suppressor immune cells at the early stages of liver colonization. **a-c** The effects of the partial deficiency of β_2_ integrin expression on the recruitment of immune cell populations 24 h after i.s. tumor cell inoculation was quantified. Immune cell subsets recruited to the liver were immuhistochemically analyzed by labeling with specific antibodies against CD11b and Ly6G antigens (CD11b^+^Ly6G^+^ cells) (**a**), and CD8 (CD8^+^ T cells) (**b**) and CD4 (CD4^+^ T cells) (**c**) lymphocytic markers. The quantification of CD4^+^, CD8^**+**^, CD11b^+^ and Ly6G^+^ (Gr1^+^) cell numbers was carried out in 3 different sections per mice, and at least 5 mice per group were used per each experiment and each one was performed 3 times. Data are mean values ± SD from 10 different fields/100 mm^2^ liver tissue section. Changes were considered statistically significant at *p < 0.05

Liver sections collected from mice bearing either C26 or β_2_-C26 cells for 24 h were immunohistochemically analyzed, and the numbers of immune cells were quantified. As shown in Fig. 8a, the infiltration of the liver by CD11b^+^Ly6G^+^ cells was significantly reduced (~ 40%) as were the numbers of CD8^+^ cells (30%)(Fig. 8b) in mice injected with β_2_-C26 cells vs. C26 cells. At this early stage of the metastatic disease, no significant differences were observed in CD4-expressing cells (Fig. 8c).

### Diminished expression of tumor β_2_ is related to a reduced infiltration of immune cell at late stages of metastatic progression

**Fig. 9:**
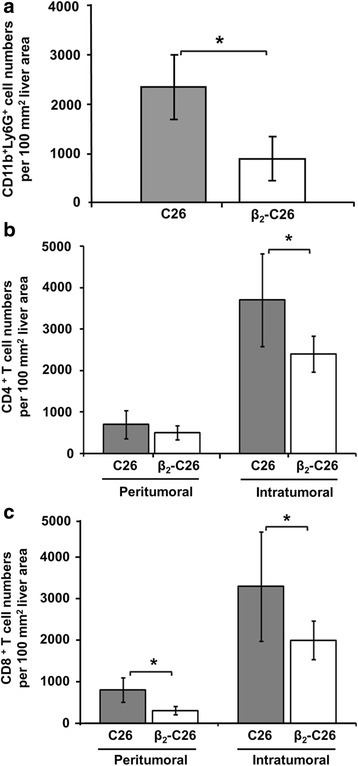
The impaired β_2_ expression on tumor cells correlates with the reduced recruitment of CD11b^+^Ly6G^+^, CD4^+^T lymphocytes and CD8^+^ T lymphocytes at late stages of C26 cells colonization of the liver. **a** The recruitment of CD11b^+^ and Ly6G^+^ (**a**), CD4^+^ T cells (**b**) and CD8^+^ T cells (**c**) to the liver was immuhistochemically analyzed 14 days after i.s. inoculation of C26 cells. The quantification of CD4^+^, CD8^**+**^, CD11b^+^ and Ly6G^+^ (Gr1^+^) cell numbers was carried out in 3 different sections per mice, and at least 5 mice per group were used per each experiment and each one was performed 3 times. Data are mean values ± SD from 10 different fields/100 mm^2^ liver tissue section. Changes were considered statistically significant at *p < 0.05 and at least 5 mice per group were used per each experiment and each one was performed 3 times

After 14 days of cell inoculation, the numbers of CD11b^+^Ly6G^+^ cells were significantly increased in tumor bearing mice comparing to the numbers observed 24 h after tumor inoculation (Fig. 9a and Additional file 3). However, this increase was weaker (by 60%) in mice inoculated with β_2_-C26 cells.

Moreover, while the numbers of the CD4^+^cell subset remained unchanged in the peritumoral areas surrounding the tumor foci, CD4^+^ T cell counts increased within the tumor foci (intra-tumoral) developed in the liver collected from mice inoculated with C26 cells but not in those tumors observed within the livers of mice bearing β_2_-C26 cells (Fig. 9b and Additional file 4). Interestingly, the numbers of CD4^+^ T cells in the peritumoral tissue of livers collected in late stages of tumor progression (Fig. 9b) were not increased when compared to those present in the early stages of liver metastasis when tumor cells are being retained in the liver (Fig. 8c). Regarding to the quantification of CD8^+^ T cells, a decrease in their numbers in those tumor foci developed in mice inoculated with β_2_-C26 cells was reported (Fig. 9c and Additional file 5). In contrast to the observation made in regard to CD4^+^ T cell count in peritumoral areas, CD8^+^ T cells count did diminished in regions surrounding the tumor foci within livers inoculated with β_2_-C26 cells.

## Discussion

Expression of LFA-1 (α_L_β_2_) on lymphoid [17] and myeloid [18] tumor cells was demonstrated in the past, and more recently also on solid tumors such as colorectal cancer [4, 12]. However, the relevance of β_2_ integrin expression to metastatic spread of the latter tumors and the mechanisms by which this integrin might act through in the tumor microenvironment remain very poorly characterized. Using a stably modified murine colon carcinoma C26 cell line with a reduced expression of β_2_ (CD18) integrin (β_2_-C26 cells), we show that the reduction in functional LFA-1 decreases the metastatic development and tumor foci size when inoculated i.s. in mice. The β_2_ subunit of the integrin is needed for LFA-1 (CD11a/CD18) activity. Therefore, we aimed to investigate which processes leading to formation of metastasis were affected by a partial deficiency in β_2_ integrin. We detected that the reduction in tumor generation in vivo, observed in mice injected with β_2_-C26 cells, was accompanied by a decrease in tumor cell adhesion in vitro and a reduced retention of tumor cells in the hepatic sinusoids in vivo*.* This uncovers an important role of tumor LFA-1 in the modulation of the metastatic progression of colorectal tumor cells to the liver. Interestingly, the adhesion of C26 cells to LSEC was affected only by β_2_ partial deficiency or by anti-CD11a neutralization, but not by tumor CD11b/c nor VCAM-1 blockade on LSECs, a ligand for the integrin CD49d (α_4_β_1_), confirming a role for LFA-1 on tumor metastasis development of colorectal cancer to the liver.

The adhesion of tumor cells to the endothelium must be followed by the process of diapedesis across endothelial cells [6, 7, 14], in order to invade parenchyma and avoid cell death by anoikis [19]. After adhesion, the transmigration of tumor cells across the endothelial layer might be mediated by LFA1/ICAM-1 interaction since the cells partially depleted in β_2_-integrin showed an impaired capacity of trans-endothelial migration. The involvement of these adhesion molecules has been also observed in the transmigration of monocytes [20] and melanoma cells [6] through the endothelium. In fact, stimulation of C26 cells with sCAM-1 increased their migratory potential. The process might be promoted by the contribution of ICAM-1/LFA-1 interaction to the endothelial cell-cell separation [21]_._ Later on, tumor cells must interact with the extracellular matrix and migrate through a pathway mediated by direct contact of C26 cells with proteins which comprise this matrix [22, 23]. The reduced adhesion to and migration across collagen type I by β_2_-C26, observed in these studies, is in line with other reports linking this integrin to adhesion [24] and transmigration of leukocytes through endothelial cell layers [25]. Garnotel et al. [26] showed that type I collagen induced the phosphorylation of both α_L_ and β_2_ subunits of LFA-1 and that the activation of protein kinase C as well as the stimulation of superoxide production by polymorphonuclear neutrophils, which was abolished by the use of neutralizing antibodies [25]. Moreover, after activation the α_L_I domain favors collagen type I, the main protein of the extracellular matrix in the tumor stroma, when compared to collagen type IV [23]. Even though, other integrins such as β_1_ might be related to the activity of β_2_ integrin, we ruled out the participation in the adhesion observed in β_2_-C26 since neutralizing antibodies against β_1_ integrins could to abrogate C26 cell adhesion nor induce a higher degree of reduction in the adhesion of β_2_-C26 cells to collagen type I. In fact, the engagement of β_2_ integrins is involved in polymorphonuclear neutrophils adhesion and extravasation by maintaining an active crosstalk with β_1_ integrin [27].

Additionally to their role as receptors for components of the extracellular matrix, integrins also regulate various cellular responses such as proliferation and loss of adhesion [26, 28]. The influence of integrins in this pathway has also been reported in a model of multiple myeloma [29]. Here, we show that the reduction in β_2_ expression, linked to the LFA-1 activity, impaired tumor cell proliferation when cultured on collagen type I. Also, these cells showed a slightly slow-down in cell division. Schmidmaier et al. [29] observed that while the in vivo progression of the tumor in a myeloma model was related to high rate of proliferation of LFA-1 positive tumor cells, they only detected a slight variation, although not significant, in the levels of several cyclins [30]. However, they observed a decrease in the survival of cells treated with an LFA-1 inhibitor which might be induced by an increase in p53 and p21. In agreement with these reports, we could detect a higher number of β_2_-C26 cells in sub G1. It is noteworthy that LFA-1 activation with sICAM-1 stimulates the activity of COX-2 and the increase in PGE_2_ production by C26 tumor cells (Arteta et al., personal communication). The increase in COX-2 activity in gastric adenocarcinoma has been related to bcl-2 expression and an increase in cell survival [31]. Indeed, NS298 and celecoxib, selective cyclooxygenase-2 inhibitors, induce apoptosis by a decrease in the activity of anti-apoptotic molecule bcl-2 in LNCaP cells [32] and Akt [33] in human prostate cells.

In previous studies, we reported that the ligation of LFA-1 expressed on tumor cells with endothelial ICAM-1 induced the production of ManR-stimulating factors from C26 cells, including IL-1, VEGF and sICAM-1 [4, 12], mediated by the activity of COX-2 in tumor cells. This up-regulation of ManR activity on LSECs was related to the inhibition of the cytotoxic activity of LSLs against the tumor after their interaction with tumor activated LSECs [4]. We found that β_2_-C26 cells were unable to increase the activity of this endothelial receptor and, as a result, the cytotoxic potential of LSLs towards tumor cells in vitro was not diminished. It is interesting to note that, while sICAM-1 stimulation of C26 cells induced the production of ManR stimulating factors, this was abolished by pre-treatment of tumor cells with either LFA-1 neutralizing antibodies or the COX-2 inhibitor celecoxib (Arteta et al., personal communication). During the immune response mounted to fight the tumor cells, different subpopulations of immune cells are recruited to the organ to be colonized. Among them, CD11b^+^ Ly6G^+^ cells, a subset of myeloid derived suppressor cells (MDSCs) [34], are recruited from the circulation to the liver attracted by an array of soluble factors derived from the liver tumor microenvironment [35]. The recruitment of CD11b^+^ Ly6G^+^ cells *(*granulocytic-MDSCs) in vivo was decreased in the liver of β_2_-C26 bearing mice. These cells are known to compromise antitumor immune surveillance especially in cancer [36, 37], preventing tumor escape from the immune system. Therefore, the reduced recruitment of different immune cell subsets found in β_2_-C26 bearing mice might additionally account for the impaired metastatic foci formation. However, if the reduced number of CD11b^+^ Ly6G^+^ is directly linked to the partial depletion of β_2_ subunit on tumor cells or to the disability of these cells to induce production of chemoattractants by other cell types needs further attention. Among others, hepatic stellate cells produce chemokines which attract CD11b^+^ Ly6G^+^ cells to the liver [38]. Additionally, hepatic stellate cells are activated within the tumor microenvironment by C26 cells activated by sICAM-1 (Arteta et al., personal communication) which might be a pathway modulated, as well, by the expression of β_2_ integrin on tumor cells. Some reports suggest that CD11b^+^ Ly6G^+^ might accumulate in the liver due to tumor-derived inflammatory factors such as PGE_2_ of COX-2 origin [39], VEGF [40] and IL-1β [41], released by LSECs after interacting with tumor cells via LFA-1/ICAM-1 [4, 12]. Taking this into account, we confirmed a significantly down-regulated expression of VEGF and COX-2 genes in β_2_-C26 cells after sICAM-1 activation, pointing their products as regulators of MDSC recruitment [42]. In contrast to the early phase, the CD11b^+^ Ly6G^+^ numbers increased with tumor progression in both C26 and β_2_-C26 bearing mice livers. However, this increase was significantly lower in β_2_-C26 injected mice. CD11b^+^ Ly6G^+^ were shown to block antitumor immunity by suppressing CD4^+^ and CD8^+^ T cells and inducing T regulatory cells [43]. Even though our in vitro studies show a significant impact on the LSLs cytotoxic activity which was related to the reduced expression of β_2_ on C26 cells, we could not detect any marker (e.g. foxp3) expressed by regulatory T cells in vivo. However, we did observe a decrease in CD4^+^ T cell counts at late, but not early, stages of the metastatic development. The decrease in the inflammatory soluble factors and adhesion molecule expression, together with the decrease in tumor cell number, may explain the reduced immune cell counts; and overall, the lack of a welcoming *milieu* for the invasion of the liver by cancer cells when functional LFA-1 is lacking due to the reduced expression the its β_2_ subunit. As mentioned before, the liver tumor microenvironment is a complex network of cell-cell and cell-matrix interactions in the presence of multiple soluble factors produced by the stromal, immune and tumor cells, being any of them a possible candidate to modulate the local immune response in the liver.

## Conclusions

In summary, our data shows that β_2_ expression on C26 cells mediates important processes involved in tumor progression, such as adhesion and migration, but also the viability of cancer cells, and the regulation of the associated local immune response. This likely involves a diminished cytotoxic response of LSLs, mediated by endothelial ManR, whose activity, in turn, depends on the interaction of ICAM-1 on LSEC with the tumor β_2_ integrin. This fact, together with the decreased production of pro-inflammatory factors and the down-regulated expression of suitable adhesion molecules, might account for the development of a tumor microenvironment which impairs the immune surveillance and promotes tumor progression. Therefore our findings indicate that blockage of LFA-1 on colon cancer cells might constitute a potential target for developing new therapeutical drugs for cancer treatment.

## Additional files

Additional file 1: A) Adhesion of tumor cells to inmobilized sICAM-1 and B) adhesion of cell lines with different level of β_2_ integrin expression to collagen type I. (DOCX 53 kb)

Additional file 2: (A) β_2_ neutralization reduces CRC metastatic development in the liver. (B and C) β_2_ neutralization reduces the migratory and adhesive potential of MC38 cells. (D) β_1_ neutralization does not reduce the adhesive potential of C26 cells. (DOCX 22 kb)

Additional file 3: Expression of the leukocyte markers CD11b and Ly6G on liver tissue. (DOCX 391 kb)

Additional file 4: Expression of the lymphocyte marker CD8 on liver tissue. (DOCX 261 kb)

Additional file 5: Expression of the lymphocyte markers CD4 on liver tissue. (DOCX 507 kb)
