# Supplementary material for: Decreased expression of the β2 integrin on tumor cells is associated with a reduction in liver metastasis of colorectal cancer in mice
Source: BMC Cancer. 2017 Dec 6;17:827. doi: 10.1186/s12885-017-3823-2 (PMC5718006; doi:10.1186/s12885-017-3823-2)
Supplement: Supplementary file 1 — A) Adhesion of tumor cells to inmobilized sICAM-1 and B) adhesion of cell lines with different level of β2 integrin expression to collagen type I. (DOCX 53 kb) [file 12885_2017_3823_MOESM1_ESM.docx]

**A**

**B**

**C**

**Additional file 1. A) Adhesion of tumor cells to inmobilized sICAM-1.** Cells were allowed to adhere to sICAM-1 (10 µg/ml) immobilized on 96 plates for 18 hours. After blocking, tumor cells were allowed to adhere for 30 minutes before adhesion quantification was performed as described in “Material and methods”. Data are mean values ± SD. Changes were considered statistically significant at *p<0’05. **B) Adhesion of cell lines with different level of β_2_ integrin expression to collagen type I.** The adhesion levels of two different clones with reduced expression of β_2_-integrin and of C26 cells transfected either with control siRNA or three different siRNAs specific for β_2_ integrin to collagen type I was analyzed as described in “Material and Methods”. **C) Effect of β_1_ integrin neutralization in the adhesion to collagen type I.** The adhesion levels of C26 cells and β_2_ integrin depleted cells after pre-treatment with neutralizing antibodie sspecific for β_1_ integrin to collagen type I was analyzed as described in “Material and Methods”.
