# Supplementary material for: Decreased expression of the β2 integrin on tumor cells is associated with a reduction in liver metastasis of colorectal cancer in mice
Source: BMC Cancer. 2017 Dec 6;17:827. doi: 10.1186/s12885-017-3823-2 (PMC5718006; doi:10.1186/s12885-017-3823-2)
Supplement: Supplementary file 2 — (A) β2 neutralization reduces CRC metastatic development in the liver. (B and C) β2 neutralization reduces the migratory and adhesive potential of MC38 cells. (D) β1 neutralization does not reduce the adhesive potential of C26 cells. (DOCX 22 kb) [file 12885_2017_3823_MOESM2_ESM.docx]

**Additional file 2.** (**A) β_2_ neutralization reduces CRC metastatic development in the liver.** Mice were sacrificed 14 days after i.s. the inoculation of either untreated MC38, or β_2_ neutralizing antibody pre-treated MC38 cells. Then, the metastatic development was quantified in paraffin embedded liver sections. Total tumor burden was quantified as the number of foci per liver tissue section. **(B and C)** **β_2_ neutralization reduces the migratory and adhesive potential of MC38 cells.** Tumor cells were added to collagen type I covered tissue culture plates or to collagen type I covered 8µm-pore membrane inserts untreated or after the treatment with β_2_ integrin specific neutralizing antibody. The migration and adhesion assays were carried as described in the section “Material and methods”. Differences were considered statistically significant at *p< 0.05.
