# Supplementary material for: Decreased expression of the β2 integrin on tumor cells is associated with a reduction in liver metastasis of colorectal cancer in mice
Source: BMC Cancer. 2017 Dec 6;17:827. doi: 10.1186/s12885-017-3823-2 (PMC5718006; doi:10.1186/s12885-017-3823-2)
Supplement: Supplementary file 3 — Expression of the leukocyte markers CD11b and Ly6G on liver tissue. (DOCX 391 kb) [file 12885_2017_3823_MOESM3_ESM.docx]

**Additional file 3. Expression of the myeloid markers CD11b and Ly6G on liver tissue**. The expression of CD11b and Ly6G was detected by labeling with specific antibodies against CD11b^+^ (green) and Ly6G^+^ (red) antigens (CD11b^+^Ly6G^+^ cells). Data are mean values ± SD from 10 different fields/liver section. Changes were considered statistically significant at *p<0’05. Scale bar 100 µm.
