# Supplementary material for: Decreased expression of the β2 integrin on tumor cells is associated with a reduction in liver metastasis of colorectal cancer in mice
Source: BMC Cancer. 2017 Dec 6;17:827. doi: 10.1186/s12885-017-3823-2 (PMC5718006; doi:10.1186/s12885-017-3823-2)
Supplement: Supplementary file 5 — Expression of the lymphocyte markers CD4 on liver tissue. (DOCX 507 kb) [file 12885_2017_3823_MOESM5_ESM.docx]

**Additional file 5. Expression of the lymphocyte marker CD4 on liver tissue.** Expression of the CD4 marker was detected in peritumoral and within the tumor foci by immunohistochemistry after labeling with specific antibodies against CD4. Secondary antibodies conjugated with Alexa-594 were used to manifest the location of CD4^+^ cells. The cells positive for CD4 expression were quantified in liver sections. Additionally, the tissue was labeled with DAPI for foci limit differentiation. Data are mean values ± SD from 10 different fields/liver section in three sections/liver. Changes were considered statistically significant at *p<0’05. Scale bar 100 µm.
